# Potential markers of preeclampsia – a review

**DOI:** 10.1186/1477-7827-7-70

**Published:** 2009-07-14

**Authors:** Simon Grill, Corinne Rusterholz, Rosanna Zanetti-Dällenbach, Sevgi Tercanli, Wolfgang Holzgreve, Sinuhe Hahn, Olav Lapaire

**Affiliations:** 1 Laboratory for Prenatal Medicine and Gynecologic Oncology, Department of Biomedicine, University Hospital of Basel, Basel, Switzerland; 2 Department of Obstetrics and Gynaecology, University Hospital of Basel, Basel, Switzerland; 3 University Hospital of Freiburg, Freiburg, Germany

## Abstract

Preeclampsia is a leading cause of maternal and fetal/neonatal mortality and morbidity worldwide. The early identification of patients with an increased risk for preeclampsia is therefore one of the most important goals in obstetrics. The availability of highly sensitive and specific physiologic and biochemical markers would allow not only the detection of patients at risk but also permit a close surveillance, an exact diagnosis, timely intervention (e.g. lung maturation), as well as simplified recruitment for future studies looking at therapeutic medications and additional prospective markers. Today, several markers may offer the potential to be used, most likely in a combinatory analysis, as predictors or diagnostic tools. We present here the current knowledge on the biology of preeclampsia and review several biochemical markers which may be used to monitor preeclampsia in a future, that, we hope, is not to distant from today.

## Background

Preeclampsia occurs in 2–5% of pregnancies in the Occident, but it complicates up to 10% of pregnancies in the developing countries, where emergency care is often inadequate or lacking. Therefore we are in need of a widely applicable and affordable test that could permit presymptomatic diagnosis in order to identify and monitor the patients at risk and thus provide the best prenatal care for these women and their child. Such a test would also be of benefit to confirm a confounding clinical diagnosis and for future studies investigating prophylactic treatments or temporizing therapies.

To be effective a screening test need to be sufficiently sensitive and specific and must provide an adequate postive predictive value [1]. Today, several promising markers have been described, alone or in combination, that might fulfill these criteria. However, these data came often from small case studies with selected populations. Therefore, there is a need for worldwile large scale prospective studies to confirm the sensitivity and specificity of these promising markers and assess their utility in different subtypes of preeclampsia before they could serve in clinically useful screening tests.

Furthermore, when evaluating new screening strategies, not only sensitivity, specificity and predictive values should be taken into account, but also costs, patient's acceptability and quality control [2]. Thus, the implementation of clinical tests will require close collaboration between the medical institutions, optimally in a worldwide network, together with the pharmacieutical industry in order to develop functional and, as best as possible, affordable tests which could profit to the pregnant women worldwide.

### Preeclampsia

Preeclampsia is a multi-system disorder of pregnancy, which is characterized by new onset hypertension (systolic and diastolic blood pressure of ≥ 140 and 90 mm Hg, respectively, on two occasions, at least 6 hours apart) and proteinuria (protein excretion of ≥ 300 mg in a 24 h urine collection, or a dipstick of ≥ 2+), that develop after 20 weeks of gestation in previously normotensive women [3,4]. Dependent on the systemic involvement, several other symptoms, such as edema, disturbance of hemostasis, renal or liver failure, and the HELLP syndrome (hemolysis, elevated liver enzymes, and low platelet counts) also complicate the clinical picture. Preeclampsia can have an early onset (preeclampsia starting before 34 weeks of gestation) or late onset (preeclampsia starting after 34 weeks of gestation), can show mild or severe symptoms (systolic blood pressure ≥ 160 mmHg or diastolic blood pressure ≥ 110 mmHg, proteinuria >5 g/24 hours, oliguria, neurological symptoms, other clinical symptoms such as deranged liver function, thrombocytopenia < 100 000 mm3, HELLP syndrome), and can evolve in eclampsia in the most severe cases. In addition, it can manifest as a maternal disorder only, with an appropriate fetal growing, or it can present itself with a growth restricted fetus (in utero growth restriction (IUGR)) or sudden fetal distress.

The disorder has a higher incidence among nulliparous women, in women who conceive with assisted reproduction techniques, and in women affected by autoimmune disorders, reflecting the probable influence of an "inexperienced" or dysregulated maternal immune system in its emergence [5,6]. On the other hand, women with pre-existing metabolic, vascular or renal disease are especially at increased risk for superimposed preeclampsia [7], possibly due to their elevated sensitivity to the mere normal physiological changes imposed by pregnancy itself.

Despite extensive clinical trials, up to date, no therapeutic approaches are available for either treatment or prevention of preeclampsia. Anti-hypertensive drugs, corticosteroids for lung maturation or magnesium sulfate to prevent from eclampsia (RCOG Guideline No. 10(A)) are given to handle (or prevent the worsening of) the symptoms and can thus temporize over the short term to allow for safe delivery with a more mature fetus. However, the maternal risks must be carefully weighted against the possible fetal benefits in temporizing management, as the risk of fatal deterioration of the maternal and/or fetal health condition is high. Several prophylactic therapies (anti-oxidant vitamins, calcium or folic acid supplementation, Aspirin) have so far failed to prove efficacious in the prevention of preeclampsia in healthy, nulliparous subjects, although some benefit has been shown in high risk groups (see [8-12] for a review on the different trials). As a consequence, the sole, though radical, resolution of preeclampsia is the removal of the placenta, and in case of prematurity, with the adverse consequence of delivering a pre-term baby. Therefore, preeclampsia, with or without IUGR, remains a major cause of maternal and neonatal mortality and morbidity worldwide.

### Placental pathophysiology in preeclampsia

The precise origin of preeclampsia remains elusive, but it is believed to be likely multifactorial. A certainty is the central role played by the placenta in its pathology [13-15]. A long standing hypothesis has been that preeclampsia develops as a consequence of some kind of immune maladaptation between the mother and the fetus during the very first weeks of pregnancy, leading to a 2-step disorder progression that can be summarized as following: in a first – asymptomatic – step, local aberrant feto-maternal immune interactions within the uterine wall lead to impaired tissue and arterial invasion by trophoblast cells. This results in failed transformation of the uterine spiral arteries and subsequently worsened placental perfusion. Chronic hypoxia or alternate periods of hypoxia/re-oxygenation within the intervillous space is expected to trigger tissue oxidative stress and increase placental apoptosis and necrosis [16,17]. the clinical disorder arises, in a second step, when the maternal vascular and immune systems cannot handle any longer the increased shedding of placentally-produced debris and the aberrant expression of pro-inflammatory, anti-angiogenic and angiogenic factors, leading to a systemic endothelial cell dysfunction and an exaggerated inflammatory response [3,18,19]. Recently, this hypothesis has been challenged [20]. It was proposed instead that intrinsic failure in trophoblast differentiation at different time points of ontogeny may lead to either a mild disorder with late-onset appearance, or IUGR complicated or not with the maternal symptoms. However, the origin of preeclampsia might not be restricted to an alteration of trophoblast differentiation, but may also in some cases depend on an underlying maternal constitutional factors such as genetic, obesity, dysfunctional maternal clearance or inflammatory systems [21].

### Potential benefits of biochemical markers in preeclampsia

Regardless of the lack of existing prophylactic and therapeutic means against preeclampsia, the search for non-invasive, blood-borne or urinary biomarkers that could predict the development or assist in the detection of this life-threatening pregnancy disorder is still of utmost importance. The availability of such markers could have decisive impact on the medical management of pregnant women and their child (e.g. refer to a tertiary centre) but also on the health costs associated with this poor medical condition. Since many years, different biophysical and biochemical markers have been investigated, based on pathophysiological observations that have been noted in case of preeclampsia, such as placental dysfunction, a generalized inflammatory response, endothelial dysfunction and activation of the coagulation system.

- Miss-diagnosis is still an issue in hospital- or community midwifery care owing to the multiple clinical symptoms associated with the syndrome [22]. The availability of one or several reliable biochemical indicators might thus help to ascertain a clinical diagnosis.

- Biochemical markers might allow the stratification of preeclamptic patients in different categories according to symptoms severity and/or pregnancy outcome and thus improve its clinical management [23].

- Very importantly, biomarkers might ensure a reliable early disease assessment in asymptomatic pregnant women, in particular among target groups at increased risk based on their clinical history (preeclampsia or hypertension in a previous pregnancy) or pre-pregnancy state (hypertension, obesity, autoimmune disease are examples of the latter).

On account of the current understanding of the etiology of this life-threatening pregnancy disorder, a major focus of research has recently been the identification of placental factors showing abnormal expression in preeclamptic placentas and the assessment of their potential use for non-invasive early prediction or early detection. On the other hand, it appears that maternally-expressed proteins may also serve this purpose. We review below a selected choice of the most promising markers that have been identified up to now (Table 1), with an emphasis on the factors which have been the subject of large-scale studies and for whom the data are not disputed.

**Table 1:** Summary of potential biochemical markers for the prediction (1. trimester, 2. trimester) or the detection (Manifest preeclampsia) of preeclampsia in maternal peripheral blood.

	**Plasma concentrations**				
**Biochemical Marker**	**1. trimester**	**2. trimester**	**Manifest** **preeclampsia**	**Reported** **combinations** **for prediction**	**Altered levels are also correlated with**
**sflt-1**	--	↑	↑	- sEng, PlGF VEGF -Ultrasound	--
**sEng**	--	↑	↑	-sflt-1, PlGF -Ultrasound	-IUGR -HELLP -SGA
**PlGF**	↓	↓	↓	-sEng, sflt-1	-SGA
**PP-13**	↓	↑	↑	-Ultrasound	-IUGR -Preterm delivery
**P-Selectin**	↑	↑	↑	Activin A, sflt-1 Other adhesion molecules	--
**Cell-free fetal DNA**	↑	↑	↑	-Inhibin-A	-IUGR -polyhydramnios -trisomy 21 -preterm labour
**Cell free DNA**	--	--	↑		
**ADAM12**	↓	--	--	--	-trisomy 21 -trisomy 18 -small for gestational age
**PTX3**	↑	↑	↑	--	-IUGR
**PAPP-A**	↓	↓	↓	--	-birthweight
**Visfatin**	--	↑↓	↑↓	--	-type-2 diabetes mellitus - gestational diabetes mellitus -obesity -IUGR
**Adreno-** **medullin**	↑	↑	↑	--	-vascular disorders

**sflt-1**: soluble fms-like tyrosine kinase 1; **sEng**: soluble Endoglin; **PlGF**: placental growth factor; **PP-13**: Placental protein 13; **ADAM12**: A disintegrin and metalloprotease 12; **PTX3**: Pentraxin 3; **PAPP-A**: pregnancy-associated plasma protein A; **IUGR**: Intrauterine growth retardation; **SGA**: Small for gestational age; **HELLP**: Hemolysis elevated liver enzymes; low platelets

## Biomarkers

### Angiogenic factors

Angiogenesis requires the complex interplay between the pro-angiogenic factors vascular endothelial growth factor (VEGF) and placental growth factor (PlGF) with their cognate receptors VEGF receptor-1 (VEGFR-1, which is alternatively called fms-like tyrosine kinase (flt)-1) and VEGFR-2 (for a review on the function of these factors: [24]). Interestingly, the placenta is a rich source of these factors [25-28]. In addition to regulating blood vessel homeostasis, VEGF, PlGF and the flt-1 receptor have been shown to be key components in regulating trophoblast cell survival and function [25,29-31].

Placental cells also secrete a soluble isoform of flt-1, which is generated through alternative splicing of the messenger RNA and acts as an anti-angiogenic factor by interacting with, and thereby neutralizing, PlGF and VEGF [32]. There is strong evidence for the occurrence of higher placental expression of sflt-1 and repeated findings of elevated circulating levels of sflt-1 and reduced free bioactive PlGF and VEGF in preeclamptic patients [18,33-37]. It was thus suggested that a part of this excess of circulatory sflt-1 may stem from the placenta.

Maternal blood levels of sflt-1 were shown to correlate with the severity of preeclampsia, whereas, in an opposite manner, the quantities of bioactive VEGF and PlGF were further decreased in patients with severe symptoms compared to normal pregnant women or preeclamptic patients with mild symptoms [18,33,38]. Alterations in sflt-1 and PlGF are also more pronounced in early onset- in comparison to late onset preeclampsia [38,39]. However, it was also shown that increased levels of sflt-1 were also associated with IUGR [40].

Remarkably, when introduced into pregnant rats, exogenous sflt-1 triggers hypertension and proteinuria, symptoms akin to those in preeclampsia [18]. Besides, condition medium of villous explants from preeclamptic placentas impaired vessel formation in vitro, which could be restored by prior immuno-depletion of sflt-1 from the conditioned medium [41]. It has therefore been proposed that the maternal endothelial dysfunction in preeclampsia was caused by the imbalance of the levels of circulatory angiogenic factors. Aberrant plasma or serum levels of sflt-1, VEGF and PlGF can indeed be measured prior the onset of the symptoms [42-47]. Increased amounts of sflt-1 are apparent in second trimester-, but not first trimester blood, in women destined to develop preeclampsia, whereas PlGF and VEGF levels already show alterations at the end of the first trimester of pregnancy in these patients. Three longitudinal studies comparing normotensive pregnancies and pregnancies with preeclampsia as an end-point, characterized the circulatory expression profile of these angiogenic factors [42,43,48]. In normotensive pregnancies, sflt-1 levels remain relatively stable until the last 2 months of gestation when they steadily increase. This increase is much more pronounced in pregnancies ending with preeclampsia and can discriminate this condition beginning approximately 5 to 8 weeks before the symptoms arise, in particular in cases with preterm (< 37 weeks) symptoms. In contrary to sflt-1, the levels of circulatory PlGF increase gradually and peak at mid gestation before declining again in uneventful pregnancies. PlGF concentration profile follows a similar pattern in women who later developed preeclampsia, however with decreased amplitude. PlGF concentrations are already significantly reduced at the end of the first trimester and remain lower throughout pregnancy. Yet, the difference in circulatory PlGF between normotensive pregnancies and those affected by preeclampsia is the highest within weeks of the onset of the clinical symptoms. As with sflt-1, the pre-symptomatic levels of circulatory PlGF seemed to correlate with the severity or time of onset of preeclampsia [49]. Urinary PlGF is likewise lower in preeclamptic patients before and at the time of symptoms [50,51].

According to some studies, the presymptomatic alterations in sflt-1 levels appeared to be specific for preeclampsia as no changes are detected in women who later deliver SGA neonate or whose pregnancies are complicated by IUGR, compared to women with normal pregnancy outcome [48,52]. However, others found that in a selected group of patients with abnormal uterine perfusion, similar alterations in sflt-1 and PlGF levels could be detected during the second trimester in cases with subsequent IUGR [53]. Nevertheless, owing to the evolving unbalance of angiogenic factors after 25 weeks of gestation in women with subsequent preeclampsia, the ratio sflt-1/PlGF has been advocated to be a reliable marker of overall preeclampsia risk. As a matter of fact, soluble flt-1 and PlGF have been launched by Roche as a screening test for preeclampsia in the second trimester in Europe and is expected to be submitted to the FDA soon.

It has recently been reported that patients with preeclampsia have lower plasma concentrations of soluble VEGF-R2 [54]. However, this biomarker may not be specific for preeclampsia as an equivalent decrease was observed in patients with SGA babies in the absence of preeclampsia.

### Soluble Endoglin

Endoglin (Eng) is a co-receptor for transforming growth factor (TGF)-β1 and TGF-β3 that is highly expressed on cellular membranes of the vascular endothelium and on the syncytiotrophoblast [55,56]. It functions as a modulator of TGF-β signaling and is involved in angiogenesis and the regulation of the vascular tone [57,58]. A circulatory form of endoglin, which consists of the extra cellular part of the molecule that may be produced through the proteo-cleavage of the placental membrane-bound form, has been identified in normal pregnancy and in preeclampsia [59]. In vitro, sEng acts as a negative regulator of angiogenesis by competitive interaction with TGF-β, thereby impairing capillary formation by endothelial cells. Furthermore, it induces high arterial pressure and vascular permeability in pregnant rats in which the protein was over-expressed. Very interestingly, the combined introduction of sEng and sflt-1 in the pregnant animals induced renal, placental and hepatic changes reminiscent of the HELLP syndrome [59].

Soluble Eng is present in substantial excess in preeclamptic patients compared to normotensive controls, and its concentrations appear to increase with the severity of the symptoms and are the highest in preeclampsia complicated by the HELLP symptom [40,59,60]. Pregnancies with IUGR without the maternal syndrome may also be characterized by elevated levels of sEng, suggesting that this factor is not specific for preeclampsia, but may be a marker for clinical conditions associated with an underlying placental pathology [40,60]. However, these results remain conflicting as others demonstrated no association between IUGR and the levels of sEng [60]. Moreover, a pilot study has suggested that sEng may prove useful in differentiating preeclampsia from other hypertensive diseases of pregnancy, such as gestational- or chronic hypertension [61]. Large scale studies will be needed in order to clarify these important issues.

Like sflt-1, sEng concentrations raise during the last 2 months of normal pregnancy. In pregnancies ending with preeclampsia, this increase occurs earlier and is steeper [48,62-65]. The distinction becomes significant starting 9–11 weeks before the clinical symptoms, for both early and late onset preeclampsia, but is more prominent for preterm preeclampsia or in women in whom preeclampsia is complicated with SGA. Altered levels of this factor throughout gestation are also associated with SGA pregnancies without the maternal symptoms [48,66]. Thus, a specific prediction can not be achieved with this analyte alone.

Several longitudinal case-control studies have therefore evaluated the potential of sEng in combination with the pro- and anti-angiogenic factors PlGF and sflt-1 for the prediction of preeclampsia [48,62,63]. The studies reported that the pattern of changes in the ratio of different combinations of these factors (PlGF/sEng; (sflt-1+sEng)/PlGF; etc), collected at 13 weeks and around 20 weeks, was more informative than the individual biomarkers at single time-point screening. One study suggested that a rigorous monitoring of the sequential changes in the profile of these three biomarkers between the first and the second trimesters permits sensitive and specific risk assessment [66]. A change in PlGF/sEng ratio that was below the median slope for controls conferred an odds ratio of 7.68 for the development of pre-term preeclampsia, and 2.46 for the development of term preeclampsia, and discriminated SGA pregnancies from preeclampsia. Further studies with large number of patients will be required to confirm these very promising preliminary results and assess the utility of analyzing these biomarkers in the clinical routine.

### P-Selectin

P-selectin is a member of the selectin family of cell surface adhesion molecules. It is expressed by platelets and endothelial cells upon activation and plays crucial roles in inflammatory reactions by supporting the recruitment and activation of circulating leucocytes, and in coagulation through the generation of leukocyte-derived "blood-borne" tissue factor [67,68]. P-selectin is rapidly shed from the cellular membrane of activated platelets and this release is suggested to contribute to most of the soluble isoform of the molecule that is found in the plasma [69].

Preeclampsia is associated with extensive platelet activation [70-72]. P-selectin-exposing micro particles with procoagulant activity, released from activated platelets, have been detected in the peripheral blood of preeclamptic women [73,74]. In addition, soluble P-selectin has been repeatedly, though not constantly, observed in higher amounts in serum or plasma of patients with this disorder [75-78].

Interestingly, it has recently been shown that alterations in the levels of soluble P-selectin before 20 weeks of gestation antedate the symptoms [79-81]. This early up-regulation of soluble P-selectin has been suggested to reflect the early but still asymptomatic disturbances of the maternal vascular system. In one of these studies, P-selectin was identified as the marker with the highest discriminatory ability among three biomolecules evaluated between gestational weeks 11 to 15 [79]. However, the combination of P-selectin with the two other markers, namely Activin A and VEGFR, showed a detection rate of only 59% (with a false-positive rate of 5%), which is not sufficient for a possible routine clinical implementation as a screening test.

### Cell-free fetal DNA

Since its detection in maternal plasma many approaches have been tested to use cell free fetal DNA for non-invasive diagnostic approaches. These include qualitative analyses like fetal sex analysis [82], determination of the fetal Rhesus status [83,84] or the analysis of fetal point mutations [85] as well as the quantitative analysis as an indicator for several fetal anomalies, e.g. fetal growth restriction [86], polyhydramnios [87], trisomy [88-90] or preterm labor [91]. The value of cffDNA in maternal plasma as an indicator for preeclampsia has first been reported by Lo et al. in a small scale study in the plasma of 20 preeclamptic women and 20 gestational age matched controls in the third trimester, where cffDNA was increased approximately 5-fold in women with preeclampsia [92]. The same effect was observed in the second trimester in a study by Zhong et al. in 10 preeclamptic women and 40 controls [93]. The so far biggest study in that field was conducted by Levine et al. with 120 preeclamptic women and 120 controls: A two- to five-fold increase of cffDNA levels was monitored starting from week 17 until three weeks before the onset of preeclampsia [94]. As the amount of fetal DNA is routinely determined by quantifying Y-chromosome specific sequences, e.g. *SRY *(sex determining region Y) and *DYS *[95], alternative approaches have been tested to overcome this limitation: An increase of total cell free DNA was observed in women with preeclampsia at term [96-98] and before the onset of preeclampsia [98]. Furthermore, approaches to analyze cffDNA independent from fetal sex, using epigenetic differences between maternal and fetal DNA have been developed, e.g. the use of the *maspin *gene, which is hypomethylated in fetal tissue [99] or the hypermethylated fetal promoter sequence of *RASSF1A *[100]. Although these approaches are promising, only one study quantifying cffDNA with the *RASFF1A *approach in 10 women with preeclampsia and 20 controls has been published [101]. cffDNA has shown some predictive value for the prediction of preeclampsia between 20–25 weeks of gestation, however, higher sensitivities and specificities can be obtained by combining several markers as has been shown in a nested case-control study for cell free DNA combined with Inhibin A in the second (n = 15 at risk for PE), n = 68 controls) and third trimester (n = 34 preeclampsia, n = 44 controls) [102]. Currently, several multicenter studies are being performed to confirm the predictive value of cffDNA to predict and monitor preeclampsia in combination with other potential markers, e.g. P-selectin, PAPP-A, PP-13, sflt-1, sEng, PlGF).

### ADAM12

ADAM12 (**a d**isintegrin **a**nd **m**etalloprotease 12) is a membrane bound zinc dependant protease and belongs to the ADAM protein family, a group of proteins involved in cell-cell and cell-matrix interactions in fertilization, muscle development and neurogenesis [103-105]. For this gene, two alternatively spliced transcripts are known, a short secreted form and a long membrane-bound form [106]. The plasma concentration of ADAM12 has been found to be altered in several pregnancy related disorders. Several studies have demonstrated that the plasma level of ADAM12 is decreased in women carrying a fetus with trisomy 21 and trisomy 18 [107-110]. It has also been shown that the ADAM12 concentration is decreased in women with other aneuploidies and in women with low for gestational age birth weights [111]. The first connection of ADAM12 serum levels to preeclampsia was demonstrated by Laigaard et al. in a study with 160 women with preeclampsia and 324 healthy controls in the first trimester [112]. The serum concentration of ADAM12 was significantly decreased in women that later developed preeclampsia. These results were confirmed by Spencer et al. in a study with two groups (1. n = 64 PE, n = 240 controls, 2: n = 24 cases, n = 144 controls) [113]. However another study failed to confirm these promising results but concluded that measurement of ADAM12 does not provide useful prediction of SGA, preeclampsia, or spontaneous preterm delivery [114].

### PP-13

Placental protein 13 (PP-13, galectin-13) was first isolated in 1983 by Bohn et al. [115,116]. It is a relatively small protein with 139 amino acids (16,118 kDa) which is highly homologous (69%) to the human eosinophil Charcot-Leyden Crystal protein, a phospholipase that belongs to the beta-galactoside binding S-type animal lectin super family. The homodimer which is linked by disulfide bonds probably has special haemostatic and immunobiological functions at the feto-maternal interface or a developmental role in the placenta [117]. The 600 bp mRNA transcript is only detectable in placental tissue but not in any other fetal or adult tissue [115,116,118,119]. The serum levels of PP-13 slowly increase during a normal pregnancy but abnormally low levels of PP-13 were detected in first trimester serum samples of women subsequently developing fetal growth restriction and preeclampsia, in particular cases with early onset [120-124]. Elevated serum concentrations of PP-13 have been found in the second and third trimester in women with preeclampsia, IUGR and in preterm delivery [123]. For this study 514 controls, 69 cases with preeclampsia, 69 cases with IUGR, 52 cases with preterm delivery and 24 cases with preeclampsia developing before 34 weeks of gestation have been included. Another study concluded that first-trimester serum levels of PP-13 may serve as a suitable marker for preterm preeclampsia but are weak for the prediction of severe preeclampsia and ineffective for mild preeclampsia at term [125].

Here again the combination of several diagnostic tools results in improved predictive power as was shown by combined measuring of first trimester serum PP-13 levels and median uterine artery pulsatility index by ultrasound. This combination achieved a detection rate for preeclampsia of 90% with a false positive rate of 6% [126]. However, this combination of serum PP-13 levels and uterine artery pulsatility index loses its predictive power when late second trimester (22–24 weeks of gestation) serum is analyzed [127]. Currently a commercial PP-13 test kit is developed for the first trimester screening for preeclampsia by Diagnostic Technologies, Haifa. The test has already been approved in Europe and approval in the United States is expected in the near future.

### PTX3

Pentraxin 3 (PTX3, tumor necrosis factor stimulated gene-14 [128]) belongs to the same family as C-reactive protein (CRP) or serum amyloid P component (SAP) and consists of 381 amino acids. The C-terminus is highly homologous to SAP and CRP whereas the N-terminus doesn't show any homology to other proteins. The according gene is organized into three exons [129] and is extremely evolutionarily conserved from horseshoe crab to human [130]. Responding to proinflammatory stimuli CRP, SAP and PTX3 are produced by various tissues. It is also expressed in tissues undergoing cell death. PTX3 then interacts with several growth factors, extra cellular matrix components and certain pathogens but is also involved in the activation of the complement system [131] and facilitates pathogen recognition by phagocytes [132]. During pregnancy, PTX3 is increasingly expressed in amniotic epithelium, chorionic mesoderm, trophoblast terminal villi, and perivascular stroma of placentae [132]. Cetin et al. and Rovere-Querini et al. showed that in case of a future preeclampsia and IUGR the PTX3 plasma levels are even more increased in all three trimesters [133,134]. So far no studies that combine PTX3 with other potential markers have been performed.

### PAPP-A

PAPP-A (pregnancy-associated plasma protein A, pappalysin 1, insulin-like growth factor binding protein-4 protease, EC 3.4.24.79) is a disulfide bond linked homodimeric peptidase of 1628 amino acids and a mass of 400 kDa [135]. It can be detected during pregnancy in maternal circulation mainly as a complex with the proform of the eosinophil major basic protein, an inhibitor of PAPP-A [136,137]. Although the reaction products are not identified yet, insulin-like growth factor binding proteins are substrates for the hydrolytic activity of PAPP-A [138]. PAPP-A is supposedly involved in local proliferative processes, for example bone remodeling [139,140]. In the recent years decreased plasma levels of PAPP-A have been reported in all trimesters in women with preeclampsia [141-150]. Furthermore, a correlation between birth weight and maternal PAPP-A plasma levels have been reported [151].

## Recent candidates

### Visfatin

Visfatin (nicotinamide phosphoribosyltransferase (Nampt) enzyme, EC 2.4.2.12) is an adipokine secreted by adipose tissue and involved in the biosynthesis of nicotinamide adenine dinucleotide as it catalyzes the condensation of nicotinamide with 5-phosphoribosyl-1-pyrophosphate to yield nicotinamide mononucleotide [152,153]. Several studies have shown that this protein shows altered plasma levels in different disorders, e.g. type-2 diabetes mellitus [154], obesity [155], fetal growth retardation [156] and gestational diabetes mellitus [157]. A recent cross sectional study by Hu et al. with 27 preeclampsia cases, 28 pregnant women in the third trimester and 28 non-pregnant women demonstrated that the maternal plasma visfatin levels were significantly decreased in women with mild preeclampsia and even more decreased in women with severe preeclampsia [158]. However, the exact opposite was reported by Fasshauer et al. [159], indicating that further, larger studies are necessary to evaluate the potential of Visfatin as a marker for preeclampsia.

### Adrenomedullin

Adrenomedullin is a peptide consisting of 52 amino acids that exhibits a long lasting hypotensive effect and was first isolated from pheochromocytoma by Kitamura et al. in 1993 [160]. It is expressed in all organs but predominantly in vascular endothelial cells and vascular smooth muscle cells where it regulates circulation and endothelial permeability [161,162]. The plasma level of adrenomedullin is elevated in several disorders, mostly in those resulting from pathologic processes in vasculature [163]. A medium scale study with 90 women (each 15 normotensive women in the first, second and third trimester, 15 women with preeclampsia between 25–38 weeks of gestation, 15 normotensive non-pregnant women and 15 hypertensive non-pregnant women) by Senna et al. showed that the plasma adrenomedullin levels increase during normal pregnancies. In preeclampsia the AM plasma levels are higher compared to normal pregnancies [164]. These results agree with the finding that elevated AM mRNA levels were found in placental tissue [165]. As AM acts as vasodilator it makes sense to assume that increased AM levels are increased to act against the preeclamptic hypertension.

### Auto antibodies against the angiotensin II type 1 (AT1) receptor

This is an old actor whose role in the pathogenesis of preeclampsia is currently being reevaluated. There is long time evidence for a role for the renin-angiotensin system in pregnancy. Exactly a decade ago, it was discovered that patients with preeclampsia develop agonistic autoantibodies against the second extracellular loop of the AT1 receptor [166]. Several in vitro studies confirmed that these antibodies can induce a profusion of responses related to hypertensive disorder in vascular cells and reduce the migration of trophoblast cells (reviewed in [167]). However, they are not specific to preeclampsia but rather mark pregnancies with abnormal uterine artery Doppler flow, such that they are also found in the circulation of normotensive women with IUGR [168]. Furthermore, they are present in recipients of unsuccessful kidney allografts who develop malignant hypertension [169]. At the time, these data suggested that the AT1 autoantibodies might be a mere secondary response to a hypoxic vascular insult. However, a recent study demonstrated a link between these autoantibodies and the overproduction of sflt-1 in human trophoblast cells or placental explants [170], raising a new interest for these molecules. Recently, a small prospective, nested, case control study has suggested that AT1 autoantibodies were common in patients with preeclampsia and, although sflt-1 was superior for early-onset preeclampsia, the AT1 autoantibodies may represent a better marker for preeclampsia developing at term [171]. Nevertheless, several questions remain to be answered before the AT1 autoantibodies could be used in a diagnosis strategy. First, no data exist as to the presence of these molecules before the pregnancy or prior the symptoms develop. Second, the detection still relies on a cumbersome bioassay, which prevents the initiation of large scale studies.

## Doppler ultrasonography of the uterine arteries

The inadequate placental perfusion has lead to the use of Doppler ultrasonography to assess the velocity of the blood flow in the uterine arteries. A persistence of an early diastolic notch after 24 weeks of gestation or abnormal flow velocity ratio's has been associated with an inadequate trophoblast invasion. Pregnancies associated with an abnormal uterine Doppler after 24 weeks of gestation (high pulsatility index and/or presence of an early diastolic notch) are associated with a more than six fold increase in the rate of preeclampsia [172].

Among high-risk patients with a previous preeclampsia, doppler ultrasound of the uterine arteries has an excellent negative predictive value, thus it is an important tool in patient management and care which is of paramount benefit for patients with PE in a previous pregnancy. However effort must be made so that this sophisticated technology becomes available in centers throughout underdeveloped countries.

A recently published systematic review assessed the use of Doppler ultrasonography in case of preeclampsia [173]. A total of 74 studies (69 cohort studies, 3 randomized controlled trials and 2 case-control studies with a total of 79'547 patients, of whom 2498 developed preeclampsia, were included. The authors showed that Doppler ultrasonography of the uterine arteries were less accurate in the first trimester, than in the second trimester. The combined data showed that the pulsatility index, alone or in combination with a persistent notching after 24 weeks of gestation is the most predictive parameter of Doppler ultrasonography to predict preeclampsia. This parameter may be used, especially in combination with other biomarkers.

Current data do not support the use of Doppler ultrasonography for routine screening of patients for preeclampsia [174]. However several studies show that the combination of the measurement of uterine perfusion in the second trimester and analysis of angiogenic markers have a high detection rate, especially for early onset preeclampsia [53,65,175].

## Identification of novel biomarkers

Although a panel of promising biomarkers already exists, a lot of effort is made to find novel candidates that bear a greater potential to identify women at risk for preeclampsia, in order to provide the best possible care for these mothers and children. Several approaches for the identification of novel potential biomarkers can be applied. Microarrays offer the possibility to rapidly screen the placental transcriptome for up- and down-regulated transcripts in preeclamptic samples compared to healthy controls hoping that the resulting proteins are excreted and detectable in maternal plasma. Comparative transcription analyses have been performed to some extent by several groups [17,176-183], although those groups were studying the molecular mechanisms of preeclampsia rather than potential biomarkers. Recently, the microarray based screening for RNA molecules in maternal circulation that are transcribed in placenta but not maternally has been reported as a potential source for pregnancy related biomarkers [184-187], including preeclampsia [188]. As the microarray technology is evolving rapidly regarding feature size and the number of available and well characterized genes, these experiments are performed continuously.

A more direct approach is to compare the proteome in the maternal circulation as transcriptome and proteome do not comply with each other. To cope with the complexity of human plasma, high throughput methods need to be employed. The classic approach is comparative 2D-gelelectrophoresis of albumin depleted plasma and subsequent mass spectrometric analysis of the remaining proteins that show different quantities [189-191]. This approach is currently being replaced by a mass spectrometric technique based on the iTRAQ^® ^reagent by Applied Biosystems [192]. Up to eight conditions can be simultaneously compared by labeling them with defined mass-tags. After fragmentation, mass analysis, identification and quantification the mass-tags allow the allocation of the proteins to each sample-pool. Therefore, this technique allows a global quantitative comparison of complex body fluids like plasma [192-195].

A new direction of research in the field of complex diseases is metabolomics, which consists in the global analysis of endogenous and secreted metabolites in a biological system. The first reports investigating the placental metabolome under varying oxygen tensions or in plasma of preeclamptic patients revealed novel redox biomarkers and suggest that this technology might bring new insights into placental function [196,197].

However, the different -omics profiling techniques are not suited for simple, low-cost, and rapid routine clinical screenings. Their use is primarily for the identification of novel biomarkers within global analyte groups. The next step is then to develop straightforward methods specifically designed for use in a hospital environment.

## Conclusion

Despite there exists many different potential markers for preeclampsia, the reliability of these markers in predicting preeclampsia has been inconsistent between different studies. Furthermore, preeclampsia is a multifaceted disorder, certain say it is not one but several diseases. Therefore, there is a need for high quality, large scale multi-center trials which enroll patients with different risks of developing the syndrome and throughout multi-ethnical background, in order to assess the predictive value of different markers and finally propose the best marker combination for a routine use in clinical settings.

## Competing interests

The authors declare that they have no competing interests.

## Authors' contributions

SG, CR and OL drafted and wrote the manuscript, RZD, ST, WH and SH: helped to draft the manuscript and gave important inputs. All authors read and approved the final manuscript.
